# Patient-derived models facilitate precision medicine in liver cancer by remodeling cell-matrix interaction

**DOI:** 10.3389/fimmu.2023.1101324

**Published:** 2023-05-04

**Authors:** Kaiwen Chen, Yanran Li, Bingran Wang, Xuehan Yan, Yiying Tao, Weizhou Song, Zhifeng Xi, Kang He, Qiang Xia

**Affiliations:** ^1^ Department of Liver Surgery, Renji Hospital, Shanghai Jiao Tong University School of Medicine, Shanghai, China; ^2^ Shanghai Engineering Research Center of Transplantation and Immunology, Shanghai, China; ^3^ Shanghai Institute of Transplantation, Shanghai, China; ^4^ Department of Gastrointestinal Surgery, Renji Hospital, Shanghai Jiao Tong University School of Medicine, Shanghai, China; ^5^ Department of Anesthesiology, Renji Hospital, Shanghai Jiao Tong University School of Medicine, Shanghai, China; ^6^ Ottawa-Shanghai Joint School of Medicine, Shanghai Jiao Tong University School of Medicine, Shanghai, China

**Keywords:** patient derived models, tumor models, cell-matrix interaction, liver cancer, precision medicine

## Abstract

Liver cancer is an aggressive tumor originating in the liver with a dismal prognosis. Current evidence suggests that liver cancer is the fifth most prevalent cancer worldwide and the second most deadly type of malignancy. Tumor heterogeneity accounts for the differences in drug responses among patients, emphasizing the importance of precision medicine. Patient-derived models of cancer are widely used preclinical models to study precision medicine since they preserve tumor heterogeneity *ex vivo* in the study of many cancers. Patient-derived models preserving cell-cell and cell-matrix interactions better recapitulate *in vivo* conditions, including patient-derived xenografts (PDXs), induced pluripotent stem cells (iPSCs), precision-cut liver slices (PCLSs), patient-derived organoids (PDOs), and patient-derived tumor spheroids (PDTSs). In this review, we provide a comprehensive overview of the different modalities used to establish preclinical models for precision medicine in liver cancer.

## 1 Introduction

Liver cancer is a highly aggressive tumor that develops in the liver and often results in poor outcomes. It has been established that liver cancer is the fifth most prevalent cancer and the second most fatal malignancy worldwide (1). Primary liver cancer (PLC) includes various heterogenic types, with hepatocellular carcinoma (HCC) covering 80% of all PLCs and intrahepatic cholangiocarcinoma (iCCA) representing most of the remaining 20%.

Notwithstanding that significant progress has been achieved over the past decade, liver cancer remains one of the most difficult cancers to treat. Currently, the most widely acknowledged clinical staging system for liver cancer staging, prognosis and treatment is the Barcelona Clinic Liver Cancer (BCLC) system, which stratifies patients from BCLC stage 0 to BCLC stage D according to tumor burden, liver function and individual physical status (2). During early stages, HCC can be surgically resected or treated with other locoregional therapies such as trans-arterial chemoembolization (TACE), local ablation with radiofrequency (RFA), and liver transplantation. However, for patients who have received HCC ablation or resection, the disease recurrence rate within 5 years is up to 70% (3). Furthermore, most HCC patients present with advanced-stage disease at diagnosis, whereby all locoregional therapies are ineffective or not feasible (4). In such cases, systemic treatment is indicated. Over 50% of patients with HCC eventually receive systemic treatments, including tyrosine kinase inhibitors (sorafenib, lenvatinib, regorafenib, etc.), immune-checkpoint inhibitors (nivolumab, atezolizumab, etc.), cytotoxic chemotherapy (single agent doxorubicin), and oncolytic virus therapy (Pexa-Vec, Telomelysin) (5). According to the 2022 updated BCLC strategy, the first-line drugs are Atezolizumab-Bevacizumab/Durvalumab-Tremelimumab. Otherwise, Sorafenib, Lenvatinib or Durvalumab may be selected based on the patient’s clinical, biochemical and radiological profile. Immune checkpoint inhibitors have yielded promising results in clinical trials (6), and molecularly targeted therapies in combination with immunotherapies are currently under study to identify their synergetic effect.

The transition from laboratory research to clinical applications is critical to achieving further success in these strategies, which highly relies on patient stratification by biomarkers. Nevertheless, due to the heterogeneity of PLC, no robust prognostic biomarkers for these treatments have been discovered, which hinders progress in clinical trials and guidance on personalized medication for patients (7). Therefore, patient-derived models are essential to better recapitulate tumor heterogeneity, tumor microenvironment (TME) and immune infiltration for predicting tumor invasion, metastasis and drug response.

## 2 Patient-derived models of cancer recapitulate the tumor microenvironment, guiding patient stratification and drug selection

Patient-derived models of cancer are widely used preclinical models to study the molecular mechanism of tumorigenesis and high-throughput drug screening (8). Under the background of PLC, we first present cell line xenografts in comparison with patient-derived xenografts (PDXs) and subsequently describe a series of patient-derived models of liver cancer, including patient-derived xenografts, induced pluripotent stem cells (iPSCs), precision-cut liver slices (PCLSs), patient-derived organoids (PDOs) and patient-derived tumor spheroids (PDTSs) in the following context.

### 2.1 A brief history of patient-derived models

Various patient-derived cell lines have been built since the establishment of the HeLa cell line in 1951 (9). In 1990, the first high-throughput cancer cell line screening program National Cancer Institute 60 (NCI60), was launched and used for antitumor drug screening (10). More recently, cancer genome analysis in cell lines has played a significant role in linking cellular drug responses with genomic characteristics (11). Besides, patient-derived cell lines can be implanted in mice to establish xenograft models for *in vivo* studies.

Nevertheless, the patient-derived cell lines go through a 2D-cultured environment that selects only a rare clone of cells and fail to recapitulate inter-tumor heterogeneity and TME, calling for models with more complexity, such as PDXs and PDOs.

It is well-established that PDXs preserve the natural microenvironment and architecture of tumors by transplanting processed tumor cells to mice (12). In 1953, a study by Helene Toolan revealed the possibility of growing human tumor cells in X-irradiated mice and rats (13). In 1970, Phillips and Gazet verified that more viable patient-derived xenografts were available if the recipients were immunocompromised (14). Since then, more and more PDXs have been established, such as lung (15) and ovarian (16) models.

Organoids are 3D-cultured cell models that largely resemble the specific architecture of organs. In 2009, Hans Clevers and colleagues described the first adult stem cell-derived organoid mimicking the intestinal stem cell niche (17), established from Lgr5+ expressing mouse intestinal stem cells. They documented a culture protocol for 3D epithelial organoids, which initiated the expansion of a variety of adult stem cell-derived organoid culture protocols for the lung (18), pancreas (19), colon (20) and liver (21). PDOs can be created for most cancer subtypes, exhibiting similarities to the parental tumor histologically and genetically. Ootani and colleagues pioneered an organoid culture system for intestinal epithelium using an air-liquid interface and underlying stromal elements (22). Organoid models based on air-liquid interface cultures showed potential in testing tumor-immune interactions and were employed in modeling the effects of immunotherapy on endogenous tumor-infiltrating lymphocytes (23) and screening aerosolized drugs for non-small cell lung cancer (24).

### 2.2 Patient-derived models serve as reliable preclinical models for the study of tumorigenesis and are efficient tools for high-throughput drug screening

Preclinical models in liver cancer can be categorized into *in vivo* and *in vitro* models. *In vivo* models include cell line xenografts and patient-derived xenografts, while *in vitro* models include patient-derived cells, induced pluripotent stem cells, precision-cut liver slices, patient-derived organoids, and patient-derived tumor spheroids. Importantly, cell-line xenografts are not patient-derived and offer limited help to precision medicine, while patient-derived cells and induced pluripotent stem cells can neither preserve TME nor preserve tumor structure. Hence, we will focus on models that can recapitulate the microenvironment of the original tissue, for example, patient-derived xenografts, precision-cut liver slices, patient-derived organoids and patient-derived tumor spheroids.

#### 2.2.1 Cell line xenograft

Implantation models bring many benefits, including low cost and can be used to evaluate various HCC treatments. Implanting stable HCC cell lines into the recipient mice is widely used to establish xenograft models. Cell line xenografts can be sorted into ectopic and orthotopic models according to the implantation location.

Subcutaneous (ectopic) cell line xenograft models represent the most common and easiest model available in the studies of HCC. This model is achieved by implanting HCC cells subcutaneously into the flank of the immunodeficient mice. Immunodeficient mice commonly encompass nude mice, which lack B and T cells, and non-obese diabetic-severe combined immunodeficient mice (NOD-SCID), which have a diminished number of NK cells, NKT cells, and macrophages in addition to deficiencies in complement pathways. Although subcutaneous cell line xenograft models are relatively easy to create, facilitate the monitoring of tumor size alterations, and are effective for screening new compounds, they do not accurately replicate the tumor microenvironment, especially the immune response and immune infiltration. Furthermore, subcutaneous xenograft models infrequently exhibit the metastatic characteristics of HCC, potentially resulting in false-positive drug responses (25).

Albeit establishing an orthotopic xenograft model may be more labor-intensive than creating a subcutaneous xenograft model, it is better suited for preserving the tumor microenvironment (26, 27). Rao Q et al. evaluated four common approaches to establishing the orthotopic xenograft models, including intrahepatic implantation of syngenic tumor tissues derived from Hepa1-6 cells and intrahepatic, intrasplenic, intravenous inoculation of Hepa1-6 cells (28). C57BL6 mice were used for their ability to preserve the integrity of tumor tissues and cell-cell interactions (29, 30). Intrahepatic implantation was the optimal technique due to its 100% success rate, shortest time to tumor formation, highest metastatic rate, and ability to maintain the pathological characteristics in C57BL6 mice (28). Immunohistochemistry assays revealed localized CD3+ and Foxp3+ T lymphocyte infiltration around tumor sites compared with neighboring normal tissues in liver sections of the orthotopically implanted mice, similar to the immune environment in the HCC patients. In addition, they observed a reduction in the serum CD4+/CD8+ lymphocyte ratio, a decrease in serum IFN-γ levels, and an increase in serum IL-10 levels. These findings suggest tumor progression and the establishment of an immunosuppressive microenvironment. Yan Mingxia et al. established stable primary HCC patient-derived cell lines from established subcutaneous patient-derived xenografts with a success rate of 50% (2/4). For three years, the two cell lines stably expressed some of the HCC molecular markers detected by flow cytometry, revealing a high similarity to the original tumor with preservation of intertumoral heterogeneity (31). Therefore, the cell lines exhibited good potential for establishing cell-line xenografts that could maintain some intertumoral heterogeneity. However, a limitation of the orthotopic xenograft model was that tumor size could not be easily monitored without sacrificing the mouse. Yao et al. demonstrated a new method to orthotopically inject Hep3B cell lines into BALB/c athymic nude mice. They reported a concomitant increase in AFP levels with increased Hep3B tumor size, providing a minimally invasive method for monitoring tumor growth by measuring serum AFP levels (32).

#### 2.2.2 Patient-derived xenograft

It is well-established that patient-derived xenografts can better preserve the original tumor properties than 2D cell line culturing. PDX models are widely used to select new drugs or test new treatment strategies. PDX models are commonly established by injecting patient-derived liver tumor tissues heterotopically (subcutaneously) or orthotopically (into the liver) into the recipient mice (25). Both implanting methods have advantages and disadvantages. Subcutaneous implantation allows easy evaluation of tumor size, while orthotopic implantation provides the xenograft an environment more identical to the original tissue. To avoid rejection of the human cancer tissue, xenograft models often use immunocompromised mice as recipients.

Compared with cell-line xenografts, PDXs can better retain the tumor architecture, vasculature, stromal components and intertumoral heterogeneity (**Table 1**). Julien et al. reported a very low gene expression difference between colorectal cancer patient samples and PDXs up to the 9^th^ passage (40). Moreover, PDXs can even preserve the human immune microenvironment for 4-5 passages in the recipient mice before being replaced by the mice cells (41, 42). Liver cancer PDXs were first established in 1996, and since then, many researchers have been dedicated to exploring ways to improve the resemblance of liver cancer PDXs to the original tumor tissues (30). Xu Wei et al. achieved a success rate of 28.1% (9/32) in HCC patient-derived subcutaneous xenografts and a success rate of 16.7% (1/6) in the subsequent passages of subcutaneous xenografts derived from the first xenograft tumor (33) while retaining the original tumor pathological characteristics. Armengol et al. successfully established 5 viable PDX models from 10 surgically resected HCC tumor tissues, as reported in their study (36). To verify the human origin of the passaged xenografts, they checked the microsatellite instability of two markers, D12S95 and BAT26. Yan Mingxia and colleagues collected resected tumor tissues from 24 HCC patients, implanted them subcutaneously into NOD/SCID mice with a success rate of 20.83% (5/24), and observed an increasing tumor take rate with the passages of mice (31). They also reported a tumorigenesis rate of 100% (8/8) by establishing orthotopic PDXs in the left hepatic lobe of BALB/c nude mice. Gu et al. established a stable cohort of 65 PDXs in BALB/c athymic mice out of 254 HCC patients (36). They demonstrated that the PDXs could accurately replicate the original tumor at both histological and gene expression levels while preserving intratumoral heterogeneity. Moreover, 81.3% (26/32) of the tested models showed detectable serum AFP, suggesting that PDXs have the potential to reflect the clinical characteristics of HCC on a large scale. Bissig-choisat et al., besides reporting the preservation of histologic, genetic and biological features of liver cancer PDX, also demonstrated the preservation of metastatic behavior (34). Gao and colleagues reported an intrahepatic metastasis rate of 75%(6/8), a bone metastasis rate of 37.5%(3/8) and a lung metastasis rate of 37.5%(3/8) in their HCC PDX models (43). Due to the increasing success rate of xenograft implantation and the faithful preservation of the original tumor features in liver cancer, PDX models have become reliable tools for validating current therapies and testing new therapies. Huynh and colleagues established HCC PDX lines and found that most of the current chemotherapeutic drugs for HCC yielded little or no antineoplastic effect *in vivo (* 37). Hu et al. explored the antitumor effects of Hsp70-expressing oncolytic virus between cytokine-inducer killer (CIK) and non-CIK-infused mice using xenograft models derived from 10 HCC patients (38). A synergistic inhibitory effect was found with the co-expression of specific Hsp70 and CIK infusion.

**Table 1:** Published articles on establishment of patient-derived xenografts for primary liver cancer.

Histological Type	Mice strain	Implantation location	Success rate	Metastasis rate	Reference
HCC	BALB/c nude mice	Orthotopic implantation	14/30 (46.7%)	7/14 (50%)	(30)
HCC	NOD/SCID mice	Subcutaneous implantation	9/32 (28.1%)	N/A	(33)
HCC	NOD/SCID mice BALB/c mice	Subcutaneous implantation Orthotopic implantation	5/24 (20.83%) 8/8 (100%)	N/A N/A	(31) (31)
HCC	BALB/c nude mice	Subcutaneous implantation	65/254 (25.6%)	N/A	(34)
HCC	BALB/c mice	Subcutaneous implantation	N/A	N/A	(35)
HCC	nu/nu mice	Orthotopic implantation	5/10 (50%)	N/A	(36)
HCC	BALB/c mice	Orthotopic implantation	8/8 (100%)	Intrahepatic 6/8 (75%) Lung 3/8 (37.5%) Bone 3/8 (37.5%) Lymph node 0/8 (0%)	(37)
HCC	Male SCID mice	Subcutaneous implantation	7/8 (87.5%)	0/7 (0%)	(38)
HCC	NOD/SCID mice with HLA-I matched HSC transplantation	Subcutaneous implantation	5/5(100%)	N/A	(39)

HCC, hepatocellular carcinoma; NOD/SCID, non-obese diabetic/severe combined immunodeficiency; HSC, hematopoietic stem cell.

Yet due to the differences between humans and mice, immune infiltration levels are also different in the TME. For instance, NK cells are the most abundant in the human liver, whereas NKT cells are the most abundant in the mouse liver (35, 44). Moreover, PDX models commonly use immunocompromised mice lacking some immune cell subsets, which can hardly mimic complete immune response, lymphangiogenesis or chemokine signaling. Additionally, PDX models can only preserve the human immune microenvironment for a short time, making long-term studies difficult. Moreover, different species bear different immune-cell-specific antigens, which leads to invalid results in immune-related target site selection (45). As a result, PDX models provide limited results in discovering immune-cell-related therapies.

However, recent scientific advances, such as the immunologically humanized mouse model, can reduce the gap between the two species. The model is modified to mimic the TME by expressing human immune cells and is commonly established through transferring human hematopoietic stem cells (HSCs), or peripheral blood mononuclear cells (PBMCs) derived from umbilical cord blood, fetal liver, bone marrow, or GM-CSF mobilized peripheral blood mononuclear cells into the bone marrow of the sublethally irradiated NSG mice. After developing a functional humanized immune system, PDX can be implanted subcutaneously or orthotopically into the recipient mice, recapitulating the immune microenvironment of the original tumor. Zhao et al. successfully established humanized immunity in NSG mice with HLA-I-matched HSCs (46). They found larger tumor sizes in the humanized PDX mice than in the NSG PDX mice, indicating a possibility of the tumor transforming the immune environment to facilitate tumor growth. Besides, they observed a significant decrease in infiltration levels in major human immune cells during xenograft tumor development in humanized mice. In addition, proinflammatory cytokine (IFN-γ, IL-2, IL-18, TNF-α) and cytolytic protein (granzyme A, granulysin) levels were analyzed and showed an initial increase during the early stage of the tumor and then decreased, corresponding to the clinical features of HCC patients. Lan and colleagues developed a novel humanized mouse model (BLT) that could establish sustained human hematopoiesis and functional human immune response by co-transplanting CD34+ hematopoietic stem cells (bone marrow, B), fetal liver (L) and thymus (T) into NOD/SCID mice (39). They reported that Thy/Liv/CD34+ mice could repopulate multilineage immune cells (B cells, T cells, dendritic cells) compared with a repopulation of mostly CD3+ T cells in Thy/Liv mice. Moreover, a strong *in vivo* immune response was observed in Thy/Liv/CD34+ mice as skin xenografts were rejected. Thus, humanized PDX model provides a promising tool for studying *in vivo* HCC behaviors and human immune responses.

#### 2.2.3 Induced pluripotent stem cell

Advances in stem cell technology have contributed significantly to cancer research and *in vitro* preclinical models. In 2007, Takahashi et al. first demonstrated the generation of induced pluripotent stem cells (iPSCs) from human skin fibroblasts with 4 defined factors: Oct3/4, Sox2, Klf4, and c-Myc (47). IPSCs have a great self-renewal capacity and differentiation potential, providing a platform for studying pathogenesis, drug screening and regenerative medicine. Moreover, patient-specific iPSCs hold the potential for treating liver cancer since these cells maintain the genetic background of their donor.

According to Kim and colleagues, pluripotent liver cancer cells were produced from four HCC cell lines using the retroviral introduction of genes associated with reprogramming, which displayed distinctive colony morphology and tumor marker expression compared to the original tumors, and demonstrated pluripotency by expressing multiple marker genes for pluripotency (48). Afify and colleagues established liver cancer stem cells (CSCs) from iPSCs by culturing in a conditioned medium (CM) for the HCC cell line Huh7. This CM could replicate a microenvironment like chronic inflammation, generating CSC without genetic manipulation (49). These studies provide methods to produce liver CSCs, defined as a tumorigenic subpopulation in liver cancer, contributing to tumor metastasis and recurrence (50). In this way, researchers can depict basic and oncological features of liver CSCs and elucidate molecular mechanisms underlying CSCs development and cancer progression. The efficacy of novel therapies has also been tested with iPSC-derived liver cancer models. In a recent study, researchers knocked down p21 of human iPSCs and observed tumorigenicity during induction differentiation of iPSCs to hepatocyte-like cells. Upon combination therapy of acyclic retinoid with AKR1B10 inhibitor, hepatoma-like cells were induced into normal hepatocytes (51).

In summary, some early attempts have been made to apply iPSCs in liver cancer modeling and therapy exploration. However, as iPSCs fail to preserve the 3D architecture and local TME and liver cancers are complex diseases evolving based on versatile genetic and environmental alterations, their potential applications are limited.

#### 2.2.4 Precision-cut liver slice

Initially developed by Smith and colleagues in 1985 for toxicity testing, precision-cut liver slices have since expanded their applications beyond toxicology and are now commonly used to model chronic liver conditions (52, 53). PCLS model preparation starts by slicing tissue cylinders with a 5-8 mm diameter into a reproducible thickness, usually 250-300 μm, which allows the diffusion of nutrients and oxygen to the inner cell layer. Subsequently, the liver slices are put in continuously submerged or dynamic culture systems and can keep viable for several days in optimal conditions (54). In PCLS models, various cellular components of liver tissue are preserved, including hepatocytes, hepatic stellate, Kupffer and endothelial cells, and functionality of all liver cell types enables research on liver function from a multicellular perspective. Moreover, unlike traditional *in vitro* models, PCLSs maintain the original extracellular matrix, including proteoglycans, glycoproteins and collagens of different types with the initial alignment of cells and cell-cell and cell-matrix communications. Furthermore, PCLS models offer a promising approach for studying the process of liver carcinogenesis and may provide early predictive value for HCC diagnosis, as they preserve *in vivo* cell-cell and cell-ECM interactions on a multicellular basis, thereby bridging the gap between *in vivo* and *in vitro* models. Currently, PCLS models have been widely used to establish versatile ex-vivo models of liver disease, including ALD models, NAFLD models, viral hepatitis models and liver cancer models (55–58). Overall, PCLSs are robust and reliable tools for studying mechanisms underlying liver injury and discovering novel therapeutic strategies.

Several studies have employed precision-cut liver slices to evaluate patient-specific responses to anti-cancer therapies in primary liver cancer (**Table 2**). In 2006, Kern MA and colleagues added selective cyclooxygenase-2 inhibitor Meloxicam to a surgical resected PCLS model of HCC and confirmed the antitumor effect of COX-2 inhibition with significantly increased tumor cell apoptosis and reduced tumor proliferation. Their research demonstrated the potential of the PCLS model in analyzing apoptosis at the tissue level and assessing drug effect through direct comparison with the ‘unaffected’ non-tumorous tissue of the same patient (63). Similarly, Zhang et al. reported the application of PCLS in systematic drug screening, using a method combined with cryopreservation and improved cell viability (59). The effect and safety of oncolytic viruses have also been tested with PCLS models. Zimmermann et al. established a series of PCLS with tissue from primary and secondary liver tumors and infected them with the oncolytic measles vaccine virus (MeV). With PCLS, a multicellular model with original ECM, the penetration and spreading capabilities of MeV were measured, and comparative testing of genetically variant MeV vaccine strains was enabled, bringing hope to the preselection of oncolytic viruses for virotherapy in a patient-specific manner (61). Besides drug response prediction with PCLS, other studies emphasized the investigation of tumor immunology, inter- and intra-tumoral heterogeneity and growth properties of liver cancer (60).

**Table 2:** Published articles on establishment of patient-derived PCLS models for primary liver cancer.

Histological Type	Sample size	PCLS size	Culture condition	Days viable	Application	Reference
HCC	3	300 μm thickness, 8 mm diameter	DMEM with supplements	Up to 2 days	Test selective COX-2 inhibitor	(59)
HCC, CCA	20	200-300 μm thickness, 8 mm diameter	WEM with supplements	Up to 5 days	Individualized oncology	(60)
HCC	30	300 μm thickness, 8 mm diameter	DMEM with supplements	At least 4 days	Test the effect of Regorafenib	(61)
HCC, CCA	53	250 μm thickness, 6 mm diameter	WEM with supplements	At least 7 days	Represent malignant phenotype	(62)

HCC, hepatocellular carcinoma; CCA, cholangiocarcinoma; DMEM, Dulbecco’s-modified Eagle’s medium; WEM, William’s E medium; COX-2, cyclooxygenase-2.

Overall, PCLS provides a versatile tool for liver cancer study with advantages in preserving complex 3D architectures and cell-cell interplay compared with traditional cell cultures. Additionally, it is easier to build and capable of presenting the immune landscape in human malignancies efficiently compared with organoids and xenografts. However, there are a few drawbacks to using the PCLS platform. Though the presence and metabolism activity of various cellular components in PCLS is confirmed when established, unexpected proliferation and functional deterioration may occur during incubation. Furthermore, the slicing process during the preparation of PCLS may bring out unavoidable damage and trigger a repair and regenerative response, resulting in fibrosis during culture. Additionally, the lifespan of PCLS is rather short, and the model is non-renewable, which hinders long-term study with the platform and limits the reproducibility of experiments, calling for other patient-derived models that are easier to maintain and available for biobanking.

#### 2.2.5 Patient-derived organoids

In recent days, the development of 3D cultures has enabled the establishment of novel *in vitro* cancer models that resemble the primary tumor epithelium that they derived from genetically and phenotypically. Organoids are self-organizing 3D structures that mimic the original *in vivo* architecture of tumors and have shown promising applications in precision medicine (62). PDOs are organoids derived from patient samples collected through surgical resections or biopsies. To culture PDOs, the tumor tissues undergo physical or enzymatical disassociation first and are subsequently embedded in an extracellular matrix (ECM), growing in the specific culture media containing growth factors and/or inhibitors required by that tissue (64). The choice of biological or synthetic scaffold mimicking the ECM depends on the tissue exhibiting different porosity, permeability, surface chemistry, and mechanical characteristics (65). Through a combination of various ECM and growth factors, researchers can now mimic the native TME in PDO, including cell-cell and cell-matrix interactions, which are lost in traditional 2D cell culture. Specifically, emerging PDOs that recapitulate TME provide more accurate and versatile tools to testify existing therapies, discover new drugs, and guide personalized treatment plans in PLC.

##### 2.2.5.1 PDO in liver cancer

PDOs derived from resected specimens (66–71) or needle biopsies (72) have been established and serve as a satisfactory platform for precision medicine in primary liver cancer. Based on established culture conditions for long-term expansion of human cells derived from healthy liver tissues (66), in 2017, Laura Broutier et al. successfully established cultures from tumors derived from eight individuals with PLC representing the three most common subtypes of cancer: HCC, CCA and combined hepatocellular-cholangiocarcinoma (CHC). To avoid contamination of nontumoral tissue and supply tumor cell growth, in this new protocol, the duration of tissue digestion is prolonged, and the culture medium is adjusted from the classical isolation medium, removing R-spondin-1, Noggin and Wnt3a while adding dexamethasone and Rho kinase inhibitor. Compact histological and marker analyses showed that these liver tumoroids accurately replicated the histological features and markers of the original tumor tissue and maintained these characteristics over long-term culture. Genome-wide transcriptomic (RNAseq) analysis further validated that this culture system recapitulated and maintained the transcriptomic alterations present in the tumor subtype of each patient, laying the groundwork for establishing an association between drug resistance screening and genetic mutation landscape. In this study, 29 anti-cancer compounds were screened, and ERK inhibitor SCH772984 was identified to have a potential therapeutic effect for the first time (67). Similarly, in 2019, Ling Li and colleagues performed high-throughput drug screening in a large cohort of primary liver cancer organoid lines, discovering several pan-effective drugs worth further consideration in systemic treatment (69). This research team evaluated the efficacy of Omacetaxine in a cohort of 40 HCC PDOs in 2021 as a potential treatment option for HCC patients. Mechanistic exploration was also carried out with HCC PDOs, and the results were validated in corresponding PDX models (68). Similarly, PDOs derived from cholangiocarcinoma have been established and tested on clinically approved agents gemcitabine, sorafenib, cisplatin, and doxorubicin (71).

In addition to therapy validation and drug discovery, liver cancer PDOs have been established and applied to screening drug resistance and probing underlying mechanisms (70, 72, 73). Single-cell RNA sequencing has been employed to depict biological and transcriptomic heterogeneity, especially cancer stem cell heterogeneity in PDOs, which is pivotal to tumor progression and drug resistance (73). Moreover, PDOs have been utilized in predicting neoantigen peptides and testing the function of neoantigen peptides induced CD8s through organoid killing assay, providing new methods to guide individualized immunotherapy (74).

##### 2.2.5.2 PDO with TME in liver cancer

**Figure 1:**
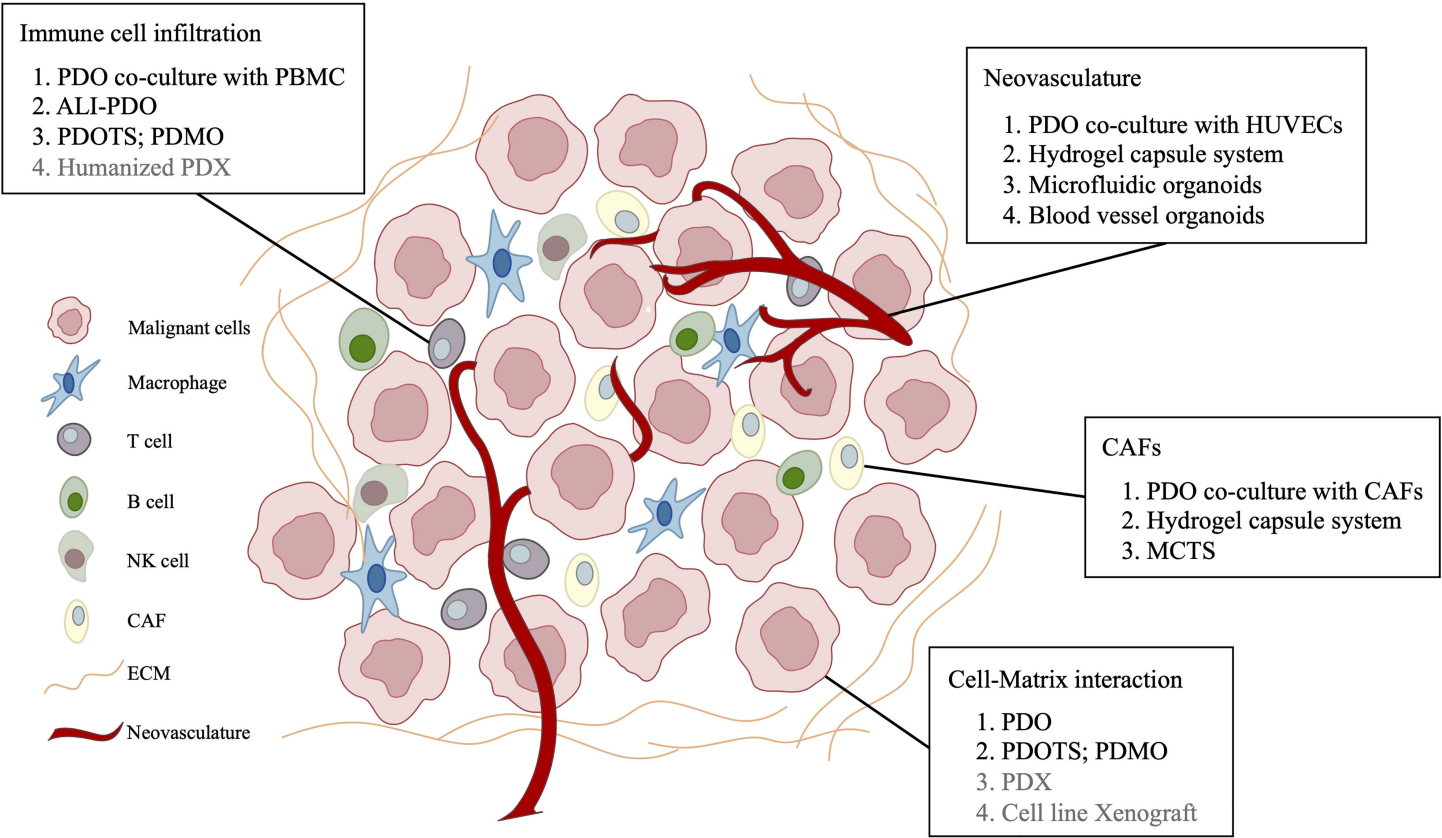
Patient-derived models remodeling cell-matrix interaction in primary liver cancer.

As mentioned above, these liver cancer PDOs have shown the potential to facilitate basic cancer research and model patient response in clinical settings. Nevertheless, they fail to preserve stromal components of primary liver cancer, which limit their capacity to serve as preclinical models. The TME, consisting of plentiful stroma, endothelial, fibroblasts, immune cells, and transformed cells, reportedly plays an essential role in cancer treatment (65). As liver cancer is generally inflammation associated, it is highly conceivable that the immunosuppressive microenvironment of liver cancer drives immune evasion and tolerance through various methods, which emphasizes the importance of target therapies. Under this context, significant endeavors have been made to establish complex liver cancer PDOs that better recapitulate TME (**Table 3**) (**Figure 1**)

**Table 3:** Published articles on establishment of patient-derived organoids for primary liver cancer.

Histological Type	Tissue collection	Success rate	Sample size	Maximum Passage	Treatment	Reference
HCC CCA CHC Normal tissue	Surgical specimen Surgical specimen Surgical specimen Biopsy	2/11 (18%) 3/4 (75%) 2/2 (100%) 3/3 (100%)	2 3 2 3	1 year	29 anticancer compounds	(68)
HCC CCA	Surgical specimen	N/A	27	N/A	129 anticancer compounds	(70)
HCC	Surgical specimen/Biopsy	N/A	40	N/A	Omacetaxine	(69)
HCC	Surgical specimen	~50%	4	N/A	Sorafenib GANT61	(71)
CCA	Surgical specimen	N/A	29	N/A	Gemcitabine, Sorafenib, Cisplatin, Doxorubicin	(72)
HCC CCA	Needle biopsy	10/38 (26%) 3/7 (43%) 45/45 (100%)	10 3 45	> 1 year	Sorafenib	(73)
HCC CCA	Surgical specimen	N/A	4 2	30	11 TKIs	(74, 75)
HCC	HCC-PDX line	14/16 (88%)	14	N/A	Sorafenib, BGJ-398	(76, 77)
HCC CCA	Surgical specimen	4/10 (40%) 2/3 (67%)	4 2	N/A	Sorafenib, Regorafenib, 5-fluorouracil	(78)
HCC CCA	Surgical specimen	18/28 (64%)	17 1	N/A	Cabazitaxel, Oxaliplatin, Sorafenib	(79)
CCA	Surgical specimen	N/A	3	N/A	N/A	(80)
HCC	Needle biopsy	N/A	3	30	HBVs-CAR-T cells, Tumor-reactive CD8+T	(81)

HCC, hepatocellular carcinoma; CCA, cholangiocarcinoma; CHC, combined HCC/CC; TKIs, tyrosine kinase inhibitor.

##### 2.2.5.3 Adding vasculature

Endothelial cells are a structural component of blood vessel conduits that deliver nutrients and oxygen to tumor tissue and participate in angiogenesis. Since most HCC tumors are hyper-vascular, angiogenesis plays a pivotal role in the pathobiology of these tumors (75). Angiogenesis is presumed to be stimulated by hypoxia, which is confirmed by decreased pO2 within HCC lesions (82). Interestingly, hypoxic conditions have been reported to suppress HIF prolyl-hydroxylase activity, leading to hypoxia-inducible factor (HIF) dimerization. Subsequently, HIFs enter the nuclei and exert their effect as a transcriptional factor, upregulating many genes, including proangiogenic factors and glycolysis-related proteins. Consequently, the disrupted balance between drivers of vessel growth and maturation (vascular endothelial growth factor, fibroblast growth factors and others) and inhibitors (angiostatin, thrombospondin-1 and others) in the microenvironment results in the proliferation of non-mature vasculatures, which are leaky, aggravating hypoxia and driving tumorigenesis (75). A global, phase 3 trial revealed that compared with those treated with sorafenib, patients treated with a combination of atezolizumab, a programmed death ligand 1 (PD-L1) inhibitor, and bevacizumab (a monoclonal antibody targeting the vascular endothelial growth factor) experienced better overall and progression-free survival outcomes, stressing the importance of endothelial components in liver TME (6). In addition, it has been shown that endothelial cells release specific angiocrine factors that promote tumor progression *via* crosstalk with cancer and other stromal cells, creating an immunosuppressive environment (83).

Attempts have been made to involve endothelial cells in patient-derived PLC models. Lim et al. successfully developed a suitable method for co-encapsulating HCC PDX-derived organoids with human umbilical vein endothelial cells (HUVECs). In their study, a HA hydrogel system comprising thiolated HA and acrylated peptides was applied to permit integrin-mediated adhesion and MMP-mediated matrix degradation, mimicking the biophysical and biochemical properties of the tumor ECM. Angiocrine factors such as MCP-1 and IL-8 were upregulated in the co-culture system, suggesting an angiocrine crosstalk between HCC and endothelial cells, which mediates expression of genes related to tumor necrosis factor signaling and directs polarized macrophages into an inflammatory and proangiogenic state (77, 83). A limitation of the study is that the HUVECs incorporated into the organoids exhibit phenotypic differences from tumor endothelial cells, and the organoids lack complex vascular structures.

Brey et al. revealed that triple co-culture systems comprising mesenchymal stromal cells and HUVECs showed a more organized development of tumor angiogenesis and vascular recruitment in breast cancer cells (84). Furthermore, microfluidic 3D culture enabled the construction of a functional circulatory system by maintaining vital parameters, a more holistic way to recapitulate vascularization in TME (85, 86). Researchers developed blood vessel organoids to simulate vessel structures in organoids, which produced vascular cells that penetrated the cerebral organoids and created a vessel-like architecture composed of CD31+ endothelial tubes (87).

##### 2.2.5.4 Adding cancer-associated fibroblasts

Cancer-associated fibroblasts (CAFs) are widely acknowledged as abundant components in a series of primary and metastatic tumor tissues, promoting cancer progression by generating extracellular matrix components and secreting versatile cytokines and nutrients, showing potential to serve as a new therapeutic target. In the HCC microenvironment, the expression of cardiotrophin-like cytokine factor 1 (CLCF1), which belongs to the IL-6 superfamily, is reportedly upregulated. CLCF1 mainly derives from CAF and induces chemokine (C-X-C motif) ligand 6 (CXCL6) and TGF- β secretion from HCC cells, which activate CAFs to express more CLCF1 through ERK1/2 signaling, generating a positive feedback loop. CLCF1-induced CXCL6 and TGF- β promote tumor progression through modulating HCC stemness. Moreover, the two cytokines facilitate the recruitment and polarization of tumor-associated neutrophils (TANs) in paracrine signaling, which exhibit potential immunosuppressive effects (88). Besides, extracellular vesicles and exosomes play an important role in CAF-mediated cancer stemness (89).

Liu et al. co-cultured 4 human CCA tumor organoids with CAFs collected and cultured from 6 human CAFs (2 of 3 CCA and 4 of 10 HCC). It has been validated that CAFs and CCA organoids enhance each other reciprocally through paracrine signaling. Additionally, through co-transplantation of tumor organoids and CAFs to mice, they demonstrated that CAFs promote CCA tumor formation and expansion *in vivo*. Sorafenib, regorafenib, and 5-fluorouracil (5-FU) treatment on these co-culture organoids showed that CAFs contribute to drug resistance, while the exact mechanism remains to be explored. However, in this study, CAFs and CCA organoids were not derived from the same patients, which may limit the ability to accurately replicate the effects of CAFs on the microenvironment (90).

In 2022, Dong and colleagues reported a more holistic suspended hydrogel capsule system to preserve stromal cells (vascular endothelial cells and CAFs) and non-cellular components, including hepatocyte growth factor (HGF), in patient-derived PLC organoids. Patient-derived tumor tissues were digested into cell clusters and added into hydrogel precursors, which contained alginate and gelatin to imitate the biomechanics of the human liver *in vivo*. Then, the hydrogel capsules were created and suspended in culture media to formulate organoids through the self-organization of the cell clusters. 18 of 28 patient-derived liver tumor organoids (64.3%) were successfully prepared. Immunofluorescence staining and whole-exome sequencing were carried out to confirm that 3D-distributed stromal components were well preserved in these PDOs in addition to molecular tumor markers and heterogeneity. Personalized drug screening was performed with several clinical anti-cancer drugs, including cabazitaxel, oxaliplatin and sorafenib, and drug response was validated with biochemical and imaging tests from a patient who received oxaliplatin, further demonstrating the promising application of this PDO establishment method to precision medicine (78).

##### 2.2.5.6 Adding immune components

Cancer immunotherapies have been under the spotlight in recent years, and versatile strategies have been applied to boost native anti-cancer immune responses or introduce exogenous immune system components that can combat tumor cells directly. Despite the success in basic medicine, the transition to the clinic remains unsatisfactory for immunotherapies, even in cancers with high mutational burdens and T-cell infiltration, emphasizing the significance of superior preclinical models (65). PDOs with immune components facilitate the progress of anti-cancer immunotherapies in several aspects, as they can be leveraged to identify combination therapies, probe novel mechanisms and predict therapeutic effects on individuals (79).

Non-parenchymal cells with immune functions are an essential component of the liver. Under normal physiological conditions, these cells maintain a delicate balance between eliminating intestinal pathogens and avoiding inflammation. However, most HCCs grow from pathologic states such as chronic liver inflammation, steatosis and fibrosis, where immune homeostasis of the liver is disrupted with increased immunosuppressive elements and elevated levels of immune checkpoint molecules (91), which impedes immune surveillance and promotes tumor progression. The immune interactions are sophisticated in liver TME. In peripheral blood and tumor tissue of patients with liver cancer, an increased level of regulatory T cells (Tregs) is found, which inhibit effector T cells through consuming IL-2, secreting inhibitory cytokines such as IL-10, TGF-β and IL-35 and expressing the co-inhibitory molecule CTLA-4 (92). Kupffer cells, macrophages residue in liver sinusoids, transform to promote immune suppression through inducing Tregs expression of PDL1 (the PD1 ligand) and other cytokines favorable for tumor progression, including TGF-β, matrix metal protease (MMP) and platelet-derived growth factor (PDGF) (93). Other immune components also shift to an immune tolerance state, such as dendritic cells, antigen-presenting cells and myeloid-derived suppressor cells (MDSCs), which can reportedly differentiate into macrophages and hinder tumor growth (94).

It has been established that immunotherapy based on immune checkpoint blockade (ICB) is a promising approach in liver cancer that has been tested in a series of clinical trials. The response rates rarely exceed 20 to 25%, with high heterogeneity in patient response (95, 96), warranting additional efforts to improve response rate and reduce ICB resistance. Moreover, the clinical benefit of ICB is not correlated with PDL1 status on tumor cells, stressing the need for alternative strategies to select candidates who may benefit from immunotherapy. Emerging PDOs of liver cancer with immune components have been reported to promote precision immune therapies in PLC patients. Zhou et al. developed a 3D co-culture model with organoids derived from CCA patients and PBMCs from healthy donors. To preserve the morphological and molecular traits of tumor organoids and immune cell infiltration, the organoids were suspended in a medium with 10% BME, supplemented with 10% human serum and nicotinamide removed OM. PBMC and purified CD3+ T cells mediated cytotoxicity was monitored, which showed high interpatient heterogeneity. Differences in transcriptomes and expression levels of immune molecules between CCA organoid lines were examined to elucidate the key to resistance to immune-mediated cell death (97). This study provides an innovative co-culture method that can be adopted to incorporate PLC organoids and patient-specific immune cells in future studies, serving as a platform to predict personalized ICB efficacy.

In addition to immune checkpoint blockade, organoids have demonstrated their potential in adoptive cell immunotherapies. Simple hepatobiliary tumor organoids without immune components have been applied to detect neoantigen peptides and elicit peptide-specific CD8+ T cells that can precisely target tumor cells (74), while co-culture systems with HCC organoids and autologous PBMC showed huge promise for CAR-T development. Zou et al. co-cultured autologous HBVs+ HCC organoids with T cells and evaluated the ability of CD39+ HBVs-CAR-T and CD39+ personalized tumor-reactive CD8+ T cells to induce apoptosis of HCC organoids, validating that CD39+ has huge potential as a biomarker for the enrichment of cytotoxic T cells and patient stratification in CAR-T therapy (80).

However, these reconstituted models could only integrate a few immune components in the original TME, limiting their ability to characterize realistic immune responses. The air-liquid interface (ALI) culturing system is a more holistic approach to immune-organoid cultures, which can maintain native stromal and immune components. The top layer of the cells is exposed to air, and the basal layer is in contact with the liquid medium, which means the organoids are cultured in a gel matrix while the lumen is exposed to air instead of submerged in media (81). Though no ALI-PDO has been reported in PLC, this technique has been widely used in other cancer types. Niklas and colleagues developed human lung and colorectal cancer organoids with ALI-PDO and found that CD45+ immune cell populations survived over 10 days (98). In another large study led by Neal, the ALI culture system was used to build organoids from different cancers. Cultures retained the tumor epithelium and its stromal microenvironment with fibroblasts and immune cells for 30 days (23).

#### 2.2.6 Patient-derived tumor spheroids

In recent years, considerable efforts have been devoted to developing alternative 3D culture methods that can overcome the limitations of organoids in onco-immune research, such as the absence of native stromal and immune cells. Moreover, the capability of PDOs in guiding clinical drug decisions is still restricted as it takes time to generate enough organoids for rapid drug screening.

Multicellular tumor spheroid (MCTS) is a 3D model whereby tumor cells grow as spherical colonies in suspension culture, with supplemented cell types to mimic the complex tumor tissue microenvironment. Compared to other preclinical models, spheroid models offer several advantages, including ease of maintenance, preservation of *in vivo* tumor growth kinetics and chemoresistance, and suitability for high-throughput drug testing (99). Yeonhwa Song and colleagues demonstrated an MCTS culture system in liver cancer, using patient-derived HCC cell lines and stromal cells from human hepatic stellate cells, human fibroblasts, and human umbilical vein endothelial cells. Drug testing revealed a clear selective response to sorafenib, 5-FU and cisplatin among MCTSs, indicating its potential role in optimizing individualized treatment (100).

In 2018, Jenkins et al. described a new 3D microfluidic patient-derived organotypic tumor spheroid (PDOTS), which preserved autologous, tumor-infiltrating immune cells. Fresh tumor specimens from patients underwent physical and enzymatic dissociation first, and spheroids ranging from 40–100 μm were filtered for subsequent PDOTS culture and profiling. The 3D microfluidic culture was enabled through a single “3D cell culture chip” presented with three self-contained microfluidic chambers. In microfluidic PDOTS, the response to PD-1 blockade and novel combination therapies was examined, suggesting that CDK4/6 inhibition augmented the response to PD-1 blockade (101, 102).

However, to guide drug selection and immune profiling in real-world clinical settings, the patient-derived model should be easy to grow from minimum tissue in a short time in addition to preservation of native TME. Shen et al. designed a novel clinical-biopsy-derived pipeline-patient-derived micro-organospheres (MOSs), which could be applied to evaluate antitumor drug response and to identify new treatment options in less than 14 days, a satisfying timeframe for guiding clinical treatment. Micro-organospheres are based on microfluidic technology and are characterized by their small size and large surface-to-volume ratio, which make T-cell infiltration into MOS less problematic. Functional analysis revealed that MOS could be used for modeling the response to personalized chemo, targeted, and radiation therapies. Moreover, the correlation between MOS assay readout and clinical outcomes has been validated in a pilot clinical trial involving eight metastatic colorectal cancer (CRC) patients (103). An automated MOS imaging pipeline combined with machine learning has been recently established, delivering on the promise of rapid tissue prototyping and high-throughput therapeutic profiling (104).

To provide a clear comparison, we summarized the advantages, disadvantages, and applications of each patient-derived model (**Table 4**).

**Table 4:** Comparison of described preclinical models for primary liver cancer.

Preclinical models	Advantages	Limitations	Applications
Cell-line xenograft	1. Easy to cultivate, high success rate 2. Low cost and time saving 3.High-throughput drug screen	1. Lack of genetic heterogeneity 2. Unable to reflect phenotype 3. Unable to simulate local TME 4. Genetic drift 5. Lack of immune cell infiltration	1. High-throughput drug screen 2. Biobank
PDX	1. Retain genetic heterogeneity 2. Simulate local TME 3. Easy to observe drug response 4. Easy to observe metastasis 5. Intact endocrine system	1. Difficulty in high-throughput screen 2. Difficulty in gene editing 3. Different genetic backgrounds 4. Time consuming 5. Lack of immune cell infiltration	1. PDXO 2. Testify novel therapy 3. Biobank 4. Nominate biomarkers
iPSC	1. Differentiation potential 2. Retain genetic background 3. Genetic manipulatable	1. Lack of 3D expansion 2. Lack of heterogeneity 3. Unable to simulate local TME	1. Liver cancer stem cells 2. Study carcinogenesis 3. Testify novel therapy
PCLS	1. 3D cancer cell growth 2. Preserve cellular components 3. Simulate local TME 4. Cultivate in short period	1. Short viable time 2. Low reproducibility 3. Altered cellular activity during incubation 4. Unavoidable fibrosis	1. Predict individualized drug response 2. Testify novel therapy 3. Study tumor behavior
PDO	1. Self-organization capacity 2. 3D cancer cell growth 3. Retain genetic background 4. Partial simulation of TME 5. Needle biopsy available	1. Failed in preserve original TME 2. Not efficient enough to guide personalized management	1. High-throughput drug screen 2. Testify novel therapy 3. Gene-response relationship 4. Biobank 5. Nominate biomarkers
PDTS	1. 3D cancer cell growth 2. Cultivate in short period 3. Simulate local TME 4. Convenience of maintenance	1. Unable to simulate systemic biological reaction 2. Low reproducibility	1. Rapid drug testing to support personalized clinical decision 2. Ex vivo profiling tumor-immune responses to ICB 3. High-throughput drug screen

PDX, patient-derived xenograft; iPSC, induced pluripotent stem cell; PCLS, precision-cut liver slice; PDO, patient-derived organoid; PDTS, patient-derived tumor spheroid; TME, tumor microenvironment; ICB, immune checkpoint blockade.

## 3 Patient-derived models facilitate precision medicine in primary liver cancer patients with unresectable tumor

Most primary liver cancer patients are diagnosed at the advanced stage, where curative surgical resection becomes infeasible. Further, the efficacy of existing systemic treatments is far from satisfactory due to high heterogeneity in liver cancer. Consequently, the clinical outcome for patients diagnosed with liver cancer remains poor, warranting preclinical models that facilitate personalized drug selection and emphasizing the need for more biomarkers that facilitate early diagnosis and patient stratification.

### 3.1 Personalized management

*In vivo* models such as PDXs have been widely used in HCC patients for high-throughput drug screening. Gu et al. established an HCC PDX cohort of 65 patients and reported a different response to sorafenib between two randomly selected groups (36). Interestingly, lenvatinib presented a better therapeutic effect compared with sorafenib. PDXs have been used in studies by Huynh et al. to search for drugs against HCC for the past few years. They established 7 HCC PDX lines and analyzed the drug response of current chemotherapeutic drugs for HCC, such as oxaliplatin, cisplatin, 17-β-estradiol, dihydrotestosterone, progesterone, EB1089, Iressa, SarCNU and doxorubicin (37). They found that oxaliplatin, cisplatin, EB1089, and Iressa yielded no antitumor effect in the PDX models. SarCNU and doxorubicin were found to significantly inhibit tumor growth, suggesting that SarCNU might be a potential drug for HCC patients. Further, SarCNU effectiveness was associated with the upregulation of P53, P21^Cip1/Waf1^ and phosphorylated cdc2 at Thy^15^, indicating that PDX can be used for drug screening and drug mechanism studies.

As for *in vitro* models, PCLSs preserve the complex phenotype and heterogeneity of individual tumors and provide a reliable predictive platform for systemic treatment with regorafenib, selective COX-2 inhibitors and oncolytic viruses (59–61). Additionally, several PDOs have been applied to predicting individual drug response to sorafenib (a tyrosine kinase inhibitor approved as first-line therapy in advanced HCC) and several other anti-cancer agents (70, 71, 105). In cholangiocarcinoma PDOs developed by Maier and colleagues, therapeutic tests have been carried out on agents commonly used in CCA treatment, including sorafenib, gemcitabine, cisplatin, and doxorubicin, showing individualized response (71). Notably, Nuciforo et al. successfully generated patient-derived liver cancer organoids from tumor needle biopsy and monitored sorafenib response. Tumor biopsies are essential as most patients who receive systemic therapies are those with intermediate and advanced tumor stages, no longer candidates for surgical resection. Importantly, biopsy-based PDOs enable personalized drug treatment (72). With the inclusion of immune components, modeling personalized responses to immunotherapies has become more feasible. Zhou et al. introduced a co-culture method by implementing autologous tumor-infiltrating immune cells in CCA organoids and evaluated the cytotoxic effects of T cells on organoids, paving the way for predicting patient-specific response to ICB (97). Similarly, another T cell co-culture model assessed the efficiency of adoptive cell therapy CD39+ HBVs-CAR-T and CD39+ personalized tumor-reactive CD8+ T cells (80).

Overall, PDMs serve as reliable precision medicine platforms, allowing for drug response testing and identifying non-responders to specific therapies, sparing patients from the high costs, inefficacy, and unnecessary adverse effects of inappropriate treatments. For those non-responders, PDMs serve as drug screening panels and identify new therapy options. In addition, high-throughput drug screening applied in a large cohort of PDMs may unveil innovative treatment strategies and revolutionize treatments in primary liver cancer. Various analysis designs and tools are available to analyze large-scale PDM models, which can help in saving time and effort (76). Quanxue Li and colleagues successfully developed a DRAP toolbox using the R package to analyze, visualize, and present drug responses on patient-derived xenograft models (106). Migliardi et al. initially developed the 1×1×1 design, which involves using one mouse per model per treatment to minimize the number of mice used in PDX experiments and lower costs (107). Gao et al. extensively applied 1 mouse per model per treatment (1×1×1) design in their preclinical cancer drug studies using 1000 PDX models, proving the design practical for large-scale drug efficacy studies (108). Jessica and colleagues evaluated the relationship between four modifiable parameters and the statistical power of the large scale 1 mouse per model per treatment (1×1×1) based drug efficacy analysis. They reported that larger treatment effect size, smaller inter-mouse variation, more frequent tumor measurement and longer study duration could increase the statistical power of the drug efficacy analysis (109). Several studies have leveraged PDOs for large-scale drug testing, presenting drugs such as ERK inhibitor SCH772984 (67) and global protein synthesis inhibitor Omacetaxine (68) as potential treatment options for liver cancer. Recent progress in developing PDOs with TME has enabled more accurate drug response prediction and illustrated the function of stromal components in drug resistance, as demonstrated by Dong et al. (78) and Liu et al. (90). Taken together, PDMs provide reliable platforms for rational drug selection, repurposing, and discovery in the personalized management of primary liver cancer patients.

### 3.2 Omics of patient-derived models

HCCs develop from hepatocytes due to the gradual accumulation of multiple genomic, transcriptomic, and epigenomic changes. Each step is crucial for tumor formation, proliferation and metastasis, stressing the significance of applying sequencing technologies to HCC research. Moreover, mass spectrum (MS) analyses provide additional information on proteomics and metabolomics, facilitating a more comprehensive interrogation of molecular characteristics of HCC. Surgical specimens are the most commonly used tissues for sequencing and MS analyses, but they only offer tumor information for patients with resectable disease. Biopsies represent the only way to obtain tumor tissues for patients with early- or end-stage disease where resection is not feasible. Unfortunately, the amount of tissue directly obtained from a liver biopsy may be insufficient for subsequent studies.

Nowadays, PDMs have emerged as excellent tools for tissue expansion and further investigation, overcoming the obstacles above. Sequencing provides insights into mutational signatures, unveiling processes related to hepatocarcinogenesis (72). Whole-exome sequencing analysis, copy number analysis, and RNA sequencing were performed on several PDX lines and liver tumoroid lines, depicting their mutational landscape, tumor mutation burden (TMB) (78, 80) and validating the genomic consistency with parental tumor tissue (36, 67). Moreover, single-cell RNA sequencing offers desirable information on intratumoral and intertumoral transcriptomic heterogeneity, which is key to dissecting drug resistance mechanisms (110). Through single-cell RNA sequencing on an HCC PDO, Zhao and colleagues exhibited that constitutive activation of downstream pathways such as PI3K-Akt might result in resistance to tyrosine kinase inhibitor (73). Heterogeneity can be discovered at proteome and metabolome dimension during MS analysis. With multi-omics data integration, enrichment studies help explore key metabolism pathways in HCC and possible mechanisms under tumor heterogeneity. Zhang et al. carried out Kyoto Encyclopaedia of Genes and Genomes (KEGG) pathway enrichment on multiple dimensions and revealed that 2 KEGG pathways (PI3K-Akt signaling pathway, synthesis and degradation of ketone bodies) were strongly correlated with T cell infiltration in the HCC TME (111).

Overall, the integration of sequencing technology and mass spectrum analysis has huge promise for multi-omics analysis in patient-derived models, enabling the extraction of gene-drug relationships and the characterization of potential drug targets for future applications.

### 3.3 Biomarkers

Although alpha-fetoprotein (AFP) is commonly used as a serum biomarker for HCC detection and diagnosis, its effectiveness is restricted, and no other reliable biomarkers can facilitate precision medicine in primary liver cancer. Currently, efforts have been undertaken worldwide to identify biomarkers with data collected from PDMs.

Early attempts have been made to apply PDOs in the quest for novel biomarkers that facilitate liver cancer early detection, prognostication and therapeutic prediction. Broutier and colleagues first examined the transcriptome profiles of tumoroids, screened out differentially expressed genes, and subsequently conducted an in-depth analysis of these genes with data from the public database TCGA (67). Wang et al. reported that PDOs could play a role in patient stratification towards target therapy, as they found a correlation between Sorafenib resistance and CD44, a marker for tumor-initiating cells (70). Meanwhile, neoantigen-associated mutational patterns investigated through PDOs facilitate the development of personalized neoantigen-directed therapies, thus improving their efficiency (74).

However, additional studies involving a larger sample size are crucial to providing more reliable readouts, and the threshold set for clinical decision-making must be carefully considered before implementing biomarkers into clinical practices. A liver cancer PDX database has been developed that integrates all genomic, transcriptome, clinical and drug response data of current PDX trials in published articles and in-house liver cancer PDXs (112). The database also provides data processing and visualization tools, making it easier for potential biomarker searching. In terms of *in vitro* models, constructing living biobanks of liver cancer organoids that cover the full spectrum of disease subtypes and molecular profiling may provide a useful tool for further validation.

## 4 Discussion

In this review, we expounded on different modalities to establish preclinical models for precision medicine in liver cancer patients, emphasizing cell-line xenografts, patient-derived xenografts, induced pluripotent stem cells, precision-cut liver slices, patient-derived organoids and patient-derived tumor spheroids.

Establishing patient-derived cell lines is inefficient and involves *in vitro* 2D culturing of the cell lines, which can lead to alterations of original properties and adaptation to *in vitro* environments. In addition, the lack of stromal components is another drawback. Thus, PDXs and PDOs are more commonly used and more reliable preclinical models for liver cancer patients.

Though liver cancer PDX models can recapitulate the TME and preserve tumor behaviors and important tumor markers, problems still exist, such as a high engraftment failure rate and long latency for engraftment. The liver cancer PDX models described in the literature are limited, with only a few providing high-throughput molecular data. Developing more PDX models would enable drug response prediction in new patients. He et al. established PDXliver, a database that included current liver cancer PDX models with corresponding drug responses and comprehensive genome and transcriptome data (112). PDXliver contains 116 PDX mouse models, 65 of which came from the literature, and the rest were from the in-house PDX platform. It allows researchers to obtain gene expression levels and somatic mutations of a given gene in PDX mice. Single nucleotide mutations and copy number mutations of a gene are also available. Besides, researchers can retrieve the drug dose, frequency, duration, and response of PDX mice. Thus, PDXliver is a more efficient tool for searching for possible biomarkers and predicting drug responses. It can also be used for patient stratification according to differential drug responses and analyze potential mechanisms. A common challenge in analyzing PDX genomic data is the lack of matched non-tumor normal samples. X.Y.Woo and colleagues developed a reliable bioinformatic analysis workflow chart that accurately detects somatic alterations in PDX models using tailored public databases (113). Moreover, MiniPDX, a new patient-derived model developed by Zhang and colleagues, provides another fast and efficient antitumor drug screening method (114). Zhang et al. revealed that MiniPDX could preserve the histopathological and morphological features of the original tumor. Furthermore, the drug responses observed in MiniPDX correlated well with those in PDX (114). Yang et al. established MiniPDX models from HCC patients to select valid postoperative drugs to help with precision medicine in end-stage HCC (115). They assessed the drug response of five single-agent drugs (sorafenib, regorafenib, lenvatinib, gemcitabine, and 5Fu+oxaliplatin) in MiniPDX models derived from 28 HCC patients through the analysis of viability and proliferation of tumor cells in the removed fiber capsules. Besides, Kaplan-Meier survival analysis showed a significantly longer disease-free survival (DFS) and a longer overall survival (OS) in the MiniPDX group than the control group. In addition, they discovered a stronger antitumor effect of sorafenib and lenvatinib in patients with high VEGFR expression. Patients with high expression of P53 showed strong rejection of gemcitabine and 5-FU+oxaliplatin (115). Hence, MiniPDX has huge prospects for future application in antitumor drug selection and biomarker discovery in postoperative HCC patients.

Though significant breakthroughs have been made, the current application of PDOs in primary liver cancer precision medicine is still limited in several aspects. First of all, the majority of successfully established liver cancer PDO lines have been derived from poorly differentiated tumors (67, 72). Besides, most specimens were collected from surgical resection, while patients who are candidates for systemic treatment and benefit most from preclinical model-guided drug selection have no surgical indications. Under this context, the PDOs established from liver needle biopsies may shed light on the personalized management of these patients. On the other hand, for studying the TME in liver tumoroids, most PLC PDO models discussed above adopted traditional reconstitution approaches, co-culturing stromal components or immune cells on the foundation of submerged Matrigel culture. In contrast, incorporating versatile technologies into organoids development has shown the prospect of creating ‘holistic’ PDOs with TME in other cancer types and is speculated to revolutionize precision medicine in liver cancer. A recent study has reported an innovative acoustically assembled patient-derived cell cluster (APCC) model, which can preserve MDSCs phenotypically and functionally. In minutes, hundreds of APCCs can be aggregated from cells by incorporating a large array of 3D acoustic trappings with ECM, recapitulating tumor-immune crosstalk. On this basis, the combinational therapeutic effect of an anti-PD-1 immune checkpoint inhibitor (pembrolizumab) and a multi-kinase inhibitor targeting MDSCs (cabozantinib) was assessed (116), showing the potential of APCCs in the personalized management of patients as well as discovering novel therapeutic strategies in liver cancer. Air liquid interface culture of organoids appears to be another solution as it captures and preserves the original tumor and, more importantly, comprises a diversity of endogenous immune cells, including T cells, B cells, macrophages and NK cells. Additionally, in ALI PDO cultures, tumor-infiltrating lymphocytes (TILs) functionally activate, expand, and show responses to PD-1/PD-L1 checkpoint blockade, as demonstrated by Neal et al. (23). Compared with co-culture PDO systems, which suffer from inevitable biases in immune composition when introducing immune components, ALI PDOs better recapitulate the diversity and function of the original TME, especially the immune microenvironment. Overall, ALI culture presents a more holistic way to recapitulate TME in PDOs, bringing hope to more precise prediction of individual response to immunotherapies in PLC patients. In recent years, development in microfluidics technology has enabled precise modulation of preclinical models at multiple dimensions, including flow conditions, shear stress, nutrient supply, input-output, and geometry (117). To evaluate the efficacy of CD8+cytotoxic T lymphocyte (CTL)-mediated tumor rejection in liver cancer, Chen et al. designed a microfluidic-based platform that mimics real tumors by incorporating a tumor center, interstitial space with recruited CTLs, feeding vessel, and simulated interstitial fluid pressure (118). However, the drawback of this model is that some typical tumor features are unexpectedly altered due to the use of only 2D cultured cell lines, emphasizing the need to construct 3D culture-based microfluidic platforms in liver cancer. Organoid-on-a-chip (OOC) refers to a new group of micro-engineered 3D models that increase the uniformity and control of organoids to ensure that high-throughput screening and testing can be carried on. Microfluidic organ-on-a-chip platforms can create a more controlled environment that optimizes the supply of nutrients and oxygen and the removal of waste (119). Moreover, sensors and actuators can be integrated with microfluidic OOC devices to ensure precise monitoring and modulation (120). Several platforms that create a variety of biomimetic organ models have been reported for the lungs (121), neuronal network (122), heart (123), liver (124) and kidney (125). The OOC models can also integrate multiple tissue compartments to mimic the function of multiple organs, allowing systematic simulation of drug metabolism in the human body and proposing more accurate prediction of drug response and toxicity, which optimizes clinical decision makings (126). More importantly, OOC models simulate the systemic response to immune therapies by combining the vascular system and considering the cytotoxic effects of circulating T cells. In contrast, current co-culture PDO models only consider tumor-infiltrating immune cells. Although there are no reports on the application of the microfluidic OOC platform in PLC, implementing microfluidic approaches with better-controlled physical and chemical parameters may provide another powerful modality for future studies on precision medicine in PLC.

Bioinformatic tools and databases are increasingly important in patient-derived models of liver cancer. The proper utilization and analysis of those models require the alleviation of misuse, misclassification, cross-contamination and erroneous cancer classification of the samples. Yet genetic drift and under-detected genomic changes are inevitable in the long-term culturing of cell lines (127, 128). The conventional methods of authentication analysis, such as short tandem repeats (STR) or single-nucleotide polymorphism assays (SNPs), may exhibit limitations when dealing with large sample batches. These methods are low-throughput, labor-intensive, and fail to detect contaminations, making them monofunctional (129). Moreover, in patient-derived xenograft models, the gradual replacement of human tumor cells with mouse stromal cells during the passaging of models, along with differences in implantation sites, random dissections, and growth variations, can cause fluctuations in allele frequencies, bringing challenges for conventional STR-and SNP-based authentication methods (130). Xiaobo Chen and colleagues reported a deep next-generation sequencing (NGS)-based multifunctional assay that can authenticate, classify, and detect contaminations in patient-derived models with higher sensitivity than traditional STR- and SNP- based authentication methods. They first profiled SNP fingerprints for each cell line sample, xenograft sample and organoid sample from deep NGS sequencing with an average depth of 3000×. Additional targeted sequencing was also performed to detect mycoplasma contamination and estimate mouse-human mix ratios. They also devised new algorithms for deep NGS data processing. The assay exhibited 100% accuracy in authenticating mouse and human cell lines, xenografts and organoids. It could stably reach 2% sensitivity in contamination detection and identify intra- or interspecies contamination. In addition, interspecies contamination can be quantified. For example, the mouse percentage in human-mouse mixed xenograft models can be calculated. Moreover, it can authenticate hundreds of samples in a single run, providing a low-cost and high-throughput assay for maintaining high-quality biobanks (131).

## Author contributions

YL and KC drafted the article. XY, YT and ZX drafted figures and tables. BW, KH and QX revised it critically for intellectual content. All authors contributed to the article and approved the submitted version.
